# Motor Properties of Peripersonal Space in Humans

**DOI:** 10.1371/journal.pone.0006582

**Published:** 2009-08-11

**Authors:** Andrea Serino, Laura Annella, Alessio Avenanti

**Affiliations:** 1 Dipartimento di Psicologia, Università degli Studi di Bologna, Bologna, Italy; 2 CsrNC, Centro di studi e ricerche in Neuroscienze Cognitive, Polo Scientifico-Didattico di Cesena, Bologna, Italy; Università di Parma, Italy

## Abstract

**Background:**

A stimulus approaching the body requires fast processing and appropriate motor reactions. In monkeys, fronto-parietal networks are involved both in integrating multisensory information within a limited space surrounding the body (i.e. peripersonal space, PPS) and in action planning and execution, suggesting an overlap between sensory representations of space and motor representations of action. In the present study we investigate whether these overlapping representations also exist in the human brain.

**Methodology/Principal Findings:**

We recorded from hand muscles motor-evoked potentials (MEPs) induced by single-pulse of transcranial magnetic stimulation (TMS) after presenting an auditory stimulus either near the hand or in far space. MEPs recorded 50 ms after the near-sound onset were enhanced compared to MEPs evoked after far sounds. This near-far modulation faded at longer inter-stimulus intervals, and reversed completely for MEPs recorded 300 ms after the sound onset. At that time point, higher motor excitability was associated with far sounds. Such auditory modulation of hand motor representation was specific to a hand-centred, and not a body-centred reference frame.

**Conclusions/Significance:**

This pattern of corticospinal modulation highlights the relation between space and time in the PPS representation: an early facilitation for near stimuli may reflect immediate motor preparation, whereas, at later time intervals, motor preparation relates to distant stimuli potentially approaching the body.

## Introduction

We can immediately and physically interact with stimuli in the external world when they occur within a limited space around us, reachable by our limbs and known as the Peripersonal Space (PPS). We might want to grab an interesting object placed in front of us or to retract a part of our body from an approaching, possibly dangerous, stimulus, such as a bee buzzing around. In order to realize these basic behaviours, our brain needs to integrate visual and auditory information about the external stimulus together with tactile and proprioceptive information about our body parts, and the result of this integration needs to be transformed into an appropriate motor plan.

In the monkey, multisensory neurons in fronto-parietal areas, integrate somatosensory information about the body with visual and acoustical information within the PPS. These neurons respond both to tactile stimuli on the monkey's arm, face or torso, and to visual and acoustic stimuli presented close, but not far (i.e. at more than 30 cm) from the corresponding body part [1]–[3]. Notably, neural responses of these multisensory cells decrease as a function of stimulus distance [4]. Somatosensory and visual receptive fields (RFs) are spatially in register: if the body part where the tactile RF is anchored moves, the visual RF shifts congruently. These neurons can therefore mediate a body-part centred multisensory representation of PPS. It has been shown that such a PPS representation has not only a sensory function, but also a motor function. Electrical microstimulation of multisensory neurons evokes a wide range of motor acts mimicking normal monkey behaviour in response to potential threats [5]. Thus, in the monkey, fronto-parietal areas representing PPS link together a multisensory representation of space with a motor representation of potential acts within that space.

In humans, neuropsychological [6], [7], behavioural [8], neuroimaging [9], [10] and electroencephalography [11] studies support the existence of neural systems representing the PPS. Although sensory components of human PPS representations have been extensively investigated, information about the possible motor features of human PPS representation is meagre. In the present study we explored hand-centred modulation of auditory space in the human motor cortex.

We recorded motor-evoked potentials (MEPs) induced by TMS to left motor cortex as a measure of the excitability of the corticospinal hand motor representation. MEPs were compared when identical sounds were presented either close to the subjects hand (at 5 cm; NEAR Sounds) or in distant space (at 100 cm; FAR Sounds). NEAR sounds, but not FAR sounds evoke a representation of the PPS around the hand (see Serino et al., 2007). Thus, a differential effect on MEPs associated with NEAR sounds compared to FAR sounds would reflect a modulation of corticospinal excitability of the hand motor representation due to the PPS representation.

Effective motor reactions to stimuli approaching the body need to be fast. In monkeys' multisensory areas, both neural responses elicited by sensory stimuli and body movements evoked by electrical stimulation show typically short latencies (up to 10–30 ms) [12]. In order to study the time-course of human corticospinal motor excitability due to PPS representation, we delivered TMS pulses at four time intervals following the auditory stimuli (50, 100, 200, and 300 msec).

In a second experiment, we asked whether proprioceptive information coding hand position was critical for modulating the motor cortex during processing of NEAR and FAR auditory stimuli. Sounds were administered in the same positions as in the previous experiment, but subjects rotated their arm so that it was off to their side, pointing slightly backwards. This way, sound to head spatial distance was kept identical to Experiment 1, but both types of sound were in the far space with respect to subjects' hand. Thus if space dependent modulation of corticospinal excitability is coded in a hand-centre reference frame, in Experiment 2 MEPs associated with NEAR sounds should not be different to those associated with FAR sounds.

## Methods

### Participants

A total of 24 healthy subjects, all students from University of Bologna, took part in the study. Twelve participants were assigned to Experiment 1 (8 females, mean age 25 y, range 22–28) and 12 to Experiment 2 (7 females, mean age 25 y, range 23–28). All subjects reported no abnormalities of touch or hearing and were right-handed. All subjects gave their written informed consent to participate in the study, which was performed with approval of the University of Bologna - Department of Psychology - ethics committee and in accordance with the Declaration of Helsinki (1964).

### Transcranial Magnetic stimulation

MEPs induced by TMS were recorded from first right dorsal interosseus (FDI, in the region of the index finger) and abductor digiti minimi (ADM, in the region of the little finger) by means of a Biopac MP-150 (BIOPAC, U.S.A.) electromyograph.

EMG signals were band-pass filtered (20 Hz–1.0 kHz, sampled at 5 kHz), digitized and stored on a computer for off-line analysis. Pairs of Ag-AgCl surface electrodes were placed in a belly-tendon montage on each muscle, with further ground electrodes on the wrist. A figure-of-8 coil connected to a Magstim Rapid^2^ stimulator (Magstim, Whitland, Dyfed, U.K.) was placed over the left motor cortex. The intersection of the coil was placed tangentially to the scalp with the handle pointing backward and laterally at a 45° angle away from the midline. In this way, the current induced in the underlying neural tissue was directed approximately perpendicular to the line of the central sulcus and was optimal for trans-synaptic activation of the corticospinal pathway [13], [14].

Using a slightly suprathreshold stimulus intensity, the coil was moved over the left hemisphere to determine the scalp position from which maximal amplitude MEPs were elicited from the FDI and the ADM muscles. The optimal position of the coil was then marked on the scalp with a pen to ensure correct coil placement throughout the experiment.

Different TMS intensities may disclose different neurophysiological modulations [15], [16], since they recruit different neural population within the motor cortex [17]. We did not have any *a-priori* hypothesis about the critical TMS intensity necessary to study motor cortex modulation by PPS representation; therefore during the experiments, we used two different intensities of magnetic pulses eliciting MEPs, namely at 120% and at 140% of the resting motor threshold (rMT). The rMT was defined as the minimal intensity of the stimulator output that produced MEPs with amplitudes of at least 50 µV with 50% probability in the muscle with the higher threshold [18], which in most cases corresponded to the ADM muscle. Mean values (S.D.) of rMT were 60.2 (8.3) in Experiment 1 and 59.4 (5.01) in Experiment 2. The two motor thresholds did not differ from one another (*p* = 0.37). The absence of voluntary contractions was continuously verified by visually monitoring of the EMG signal.

### Procedure

**Figure 1:**
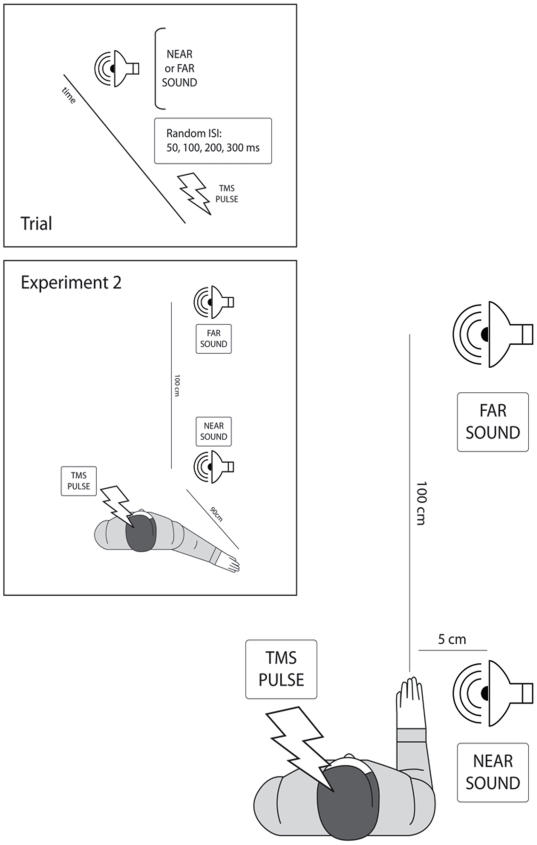
Experimental set up. The main panel represents the experimental set up and a typical subject during Experiment 1. The small upper panel represents the sequence of events in each trial. The small lower panel represents a typical subject during Experiment 2, when participants placed their right arm to the side, with the hand pointing backwards (far from the source of near sounds).

Each subject was seated on a comfortable chair with the right arm placed on an arm rest. Two identical loudspeakers were placed in front of the subject and to the right, either in a NEAR position, at ≈60 cm from the subject head, or in a FAR position, 100 cm away from the near position, thus at ≈165 cm from the subject head (see Figure 1). In Experiment 1, the subjects right hand was placed close to the NEAR loudspeaker: therefore the distance between the hand and the sound sources was ≈5 cm for the NEAR loudspeaker and ≈100 cm for the FAR loudspeaker. In Experiment 2, the subject's right arm was rotated and pointed slightly backward, and therefore the subject's right hand was placed at ≈80 cm from the NEAR loudspeaker and ≈180 cm from the FAR loudspeaker. In this way, both in Experiment 1 and in Experiment 2 the two types of auditory stimuli were close to or far from the subject's head, but only in Experiment 2 were both of them far from the hand.

Participants were blindfolded during the whole duration of the experiment and oriented their heads towards the front.

To maintain attention throughout the experimental session, subjects were requested to monitor the right hand for the infrequent occurrence of specific tactile stimuli (see below).

On each trial an auditory stimulus (NEAR or FAR) was presented and TMS-induced MEPs were simultaneously recorded from the FDI and the ADM muscles. These two muscles were chosen to explore whether the possible modulation of corticospinal excitability due to PPS representations affected the motor representation of the whole hand (FDI and ADM) or was specific for the muscle that was contiguous to the source of auditory stimulation (ADM). Indeed, in the Experiment 1 set up, the NEAR sound was closer to the ADM muscle than to the FDI muscle.

The inter-trial interval randomly varied between 10 and 12 sec. The choice of this long inter-trial interval was based on a study demonstrating that TMS pulses delivered for 1 h at 0.1 Hz frequency did not induce any change in motor excitability [17]. Subjects were instructed to ignore any auditory stimulation and to focus only on the tactile stimulation administered to their right hand during the inter-trial intervals.

In order to study the time course of the motor changes evoked by auditory stimulation, TMS pulses were given at 4 different intervals: at 50, 100, 200 and 300 ms after the sound presentations.

Thus, the overall experimental design included a random combination of 2 sound locations (NEAR and FAR) and 4 TMS Delays (50, 100, 200, 300 ms), and a blocked combination of 2 TMS Intensities (120% and 140% of rMT). Each combination was randomly repeated 12 times, resulting in a total of 192 trials distributed across 6 experimental blocks, 3 with a TMS intensity at 120% rMT and 3 with a TMS intensity at 140% rMT. The order of the blocks was randomized.

Two baseline blocks of 12 trials at 120% rMT and 140%rMT were recorded before (PRE) and after (POST) the experimental session. During the baseline trials neither auditory nor tactile stimulation occurred.

### Auditory stimulation

Inspection of phono-spectral waves, as recorded by a computerized software from the two loudspeakers, assured the sounds to be equal at their origin. Before each experimental block, the two loudspeakers were calibrated with a phonometer such that the intensity of sounds from both the NEAR and the FAR loudspeakers was identical at the subject's head (70 dB, 150 ms).

We chose this relatively low intensity to avoid inducing any startle responses in the EMG signal [19]. Indeed, loud auditory stimuli presented binaurally through headphones are known to suppress MEPs recorded after 30–60 ms from both distal and proximal muscles [20], [21], an effect likely due to cortico-reticular projections to the spinal cord. Auditory stimuli normally used to induce startle responses are quite louder (90–100 db) than those used in the present study [19]. An equal proportion of NEAR and FAR sounds was administered unpredictably.

### Tactile stimulation

Tactile stimuli were delivered via three miniaturized solenoids (M & E Solve, Rochester, UK; http://www.me-solve.co.uk), placed on the middle of the dorsal surface of the right hand at a distance of 5 mm one from each other. In different trials, either a single solenoid was briefly (5 ms) activated (weak stimulus) or all solenoids were activated together (strong stimulus): subjects had to respond, lifting the tip of their left foot, only to the strong stimulus. Tactile targets were rare, comprising 20% of total trials (equally frequently preceded by a NEAR or a FAR sound). An experimenter visually monitored subjects' responses. Tactile stimuli were administered in the inter-trial interval at least 4–5 sec apart from TMS pulses to avoid MEP contamination due to tactile stimuli or motor responses [22], [23]. Error rates (false alarm, miss) were very low (<2%) and were constant throughout the experiment.

### Data analysis

Neurophysiological data were processed off-line. Trials with EMG activity prior to TMS were discarded from the analysis (less than 5% in each subject). Mean MEP amplitude values in each condition were measured peak-to-peak (in mV).

The amplitudes of raw MEPs recorded during baseline blocks were analyzed by means of a mixed-model ANOVA, with Muscle (FDI and ADM), TMS Intensity (120% and 140% of rMT) and Session (PRE and POST) as within-subjects factors, and with Experiment (arm forwards, EXP1, and arm backwards, EXP2) as a between-subjects factor.

The MEPs evoked during both PRE and POST baselines were averaged and used to compute an index of MEP modulation (MEPi), calculated as the ratio between the averaged MEPs recorded in each experimental condition and the averaged MEPs recorded in the baseline session, multiplied by 100. In this way, a MEPi = 100% indicates no modulation, MEPi>100% indicates an enhancement and a MEPi<100% indicates a reduction of corticospinal excitability with respect to the baseline.

MEPi data were entered in a mixed-model ANOVA with Muscle (FDI, ADM), TMS Intensity (120%, 140% of rMT), Delay (50, 100, 200, 300 ms) and Space (NEAR, FAR) as within-subjects factors and Experiment (EXP1, EXP2) as a between-subjects factor. When a significant quadruple or triple interaction was found, further analyses were performed by splitting the analysis into separate ANOVAs. Greenhouse-Geisser corrections were used to overcome possible violation of Sphericity assumption [24].

## Results

The preliminary Muscle × TMS Intensity × Session × Experiment ANOVA on raw MEPs recorded during baseline blocks revealed a significant effect of TMS Intensity only (*F*_2,18_ = 45.57, *p*<0.00001). As expected, amplitudes of MEPs induced by stronger TMS pulses (140% of rMT) were higher (mean±s.e.m.: 1.42 mV±.12) than those recorded with lower TMS pulses (120% of rMT; 0.88 mV±.11). This effect was equally present in the two experiments, for both the recorded muscles, and before and after each experiment, since no significant interaction between Intensity and the other factors was found (*p_s_*>.35). Importantly, neither the main effect of Session (*p* = .35), nor any other interaction with Session were significant (*p_s_*>.38), thus indicating that the overall excitability of the corticospinal system did not change over the course of the experiments. No other effects were significant (*p_s_*>.20).

**Figure 2:**
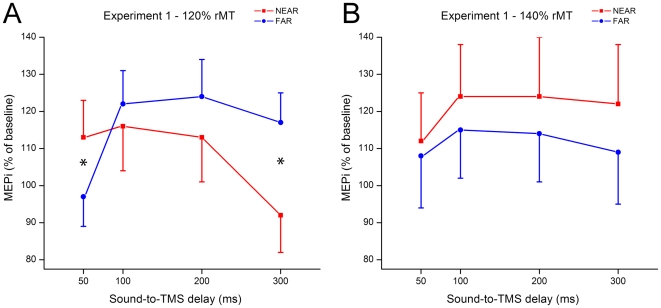
Mean MEP amplitude with respect to baseline (MEPi) recorded when sounds were presented NEAR (red lines) and FAR (blue lines) from the subjects' right hand (Experiment 1). (A) MEPi recorded with lower (120% rMT) TMS pulse intensity. (B) MEPi recorded with higher (140% rMT) TMS pulse intensity. Error bars denote s.e.m. Asterisks indicate a significant NEAR-FAR comparison (*p*<.05).

**Figure 3:**
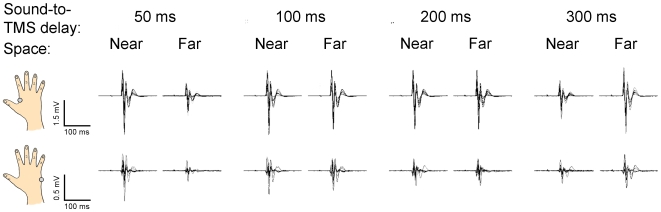
Raw MEPs amplitudes recorded from the FDI (top) and the ADM muscle (bottom) in one representative subject from Experiment 1 (only 120% rMT blocks are shown).

Baseline MEPs were averaged and used to compute an index of MEP modulation (MEPi) during the experimental session with auditory stimulation. The ANOVA on MEPis revealed a significant four-way interaction between Space, Intensity, Delay and Experiment (*F*_3,66_ = 2.76, *p*<.05). To further analyze this interaction, two separate Muscle × Space × Intensity × Delay ANOVAs were performed for each Experiment. The ANOVA run on Experiment 1 data revealed a triple Space × Intensity × Delay interaction (*F*_3,33_ = 7.40, *p*<.0008); thus we run two separate Muscle × Space × Delay ANOVAs for each Intensity. ANOVA on MEPi recorded with the lower TMS intensity (120% rMT) revealed a significant main effect of Space (*F*_1,11_ = 5.81, *p*<.04) and Time (*F*_3,33_ = 5.05, *p*<.01) and most importantly, a highly significant Space × Delay interaction (*F*_3,33_ = 7.56, *p*<.003; see Figure 2A). Post-hoc comparisons (Newman-Keuls Test) showed that MEPis recorded 50 ms after sounds occurrence were significantly enhanced when sounds were administered in the NEAR (mean MEPi±s.e.m.: 113%±9) rather than in the FAR (97%±7; *p*<.03) space. This effect disappeared when TMS pulses were administered 100 and 200 ms after sound presentations, and MEPis were not-significantly higher when FAR (122%±9 and 124%±10 for 100 ms and 200 ms of Delay respectively) rather than NEAR (116%±11 and 113%±10) sounds were presented (*p_s_*>.46). At a delay of 300 ms from sound presentation, the MEPi modulation found at 50 ms was completely reversed: at the long delay, the MEPis were significantly higher when FAR (117%±8) rather than NEAR (92%±9; *p*<.005) sounds were presented. Thus, MEPs were modulated by the presentation of NEAR and FAR sounds, and the direction of the effect depended on the time delay between MEP recording and sounds presentation. The interaction Muscle × Space × Delay was not significant (*F*_3,33_ = 0.52, *p* = .64), indicating that the two muscles were similarly modulated as a function of space and time. Examples of raw MEPs recorded from the FDI and ADM muscle in these conditions (Experiment 1, 120% rMT) are shown in Figure 3.

In Experiment 1, when TMS pulses were administered at 140% of rMT (Figure 2B), MEP amplitude values associated to NEAR auditory stimuli were numerically higher than those related to FAR stimuli (Figure 2B); however, no significant main effects, nor interactions, were found in the Muscle × Space × Delay ANOVA (*p_s_*>.14).

**Figure 4:**
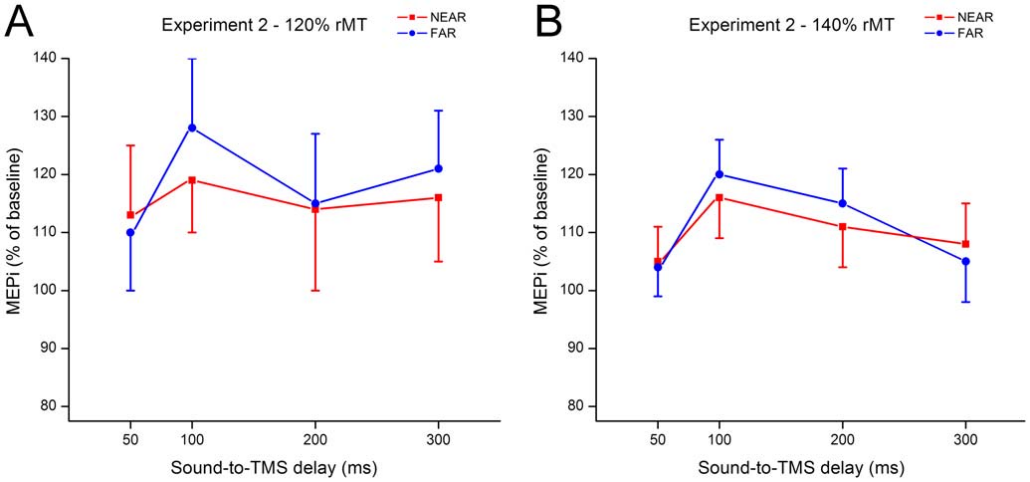
Mean MEP amplitude with respect to baseline (MEPi) recorded when sounds were presented NEAR (red lines) and FAR (blue lines) from the subjects' body (Experiment 2). (A) MEPi recorded with lower (120% rMT) TMS pulse intensity. (B) MEPi recorded with higher (140% rMT) TMS pulse intensity. Error bars denote s.e.m.

The Muscle × Space × Intensity ANOVA performed on MEPis recorded in Experiment 2 did not show any significant main effect or interaction (*p_s_*>.12). Therefore, as Figure 4 clearly shows, no relevant modulation of MEPs was recorded when participants rotated their arm backwards, thereby placing their hand quite distant from the previously NEAR loudspeaker.

## Discussion

In the present study we show for the first time that the PPS representation in humans modulates neural activity within the motor system. We used MEPs evoked by single pulse TMS to assess the excitability of the hand representation in the motor cortex during the presentation of identical task-irrelevant auditory stimuli, administered either in near or far space. Stimulus distance was defined relative to a hand-centred reference frame.

In Experiment 1 we found that an auditory stimulus presented near the hand resulted in a specific modulation of the hand motor representation in comparison with an identical stimulus presented far from the hand. This effect was intensity dependent, since the near-far difference was present with TMS pulses delivered at 120% rMT and absent with higher (140%) intensities (see below).

Crucially, the different motor modulation for near and far stimuli detected at lower TMS intensities dynamically varied as a function of time. MEPs recorded 50 ms after presenting the sound close to the hand were enhanced in comparison to when the sound was administered far from the hand. This effect faded when MEPs were recorded 100 and 200 ms after sound presentation, and it was completely reversed for MEPs recorded at 300 ms: at that time delay, sounds administered far from the hand enhanced MEPs compared to sounds administered close to the hand.

Importantly, the different effects associated with near and far sounds were linked to hand-centred reference frames [10], [25]. When subjects placed their arm backwards, thus moving the hand away from the source of near sounds, while keeping constant the distance between the sounds and the rest of their body, MEPs associated to near and far sounds were comparable. This finding suggests that hand proximity, and not head or body proximity, was critical in modulating the excitability of the hand motor representation. This finding is also important in excluding the possibility that the changes in hand corticospinal excitability found in Experiment 1 were simply due to differential levels of arousal evoked by hearing a sound near or far from the body, and it further hints at the existence of a hand-centred representation of the auditory space [25; see also 26 for a similar finding in the case of visual peri-hand space]. Furthermore, the differential effects found in Experiment 1 and in Experiment 2 also suggest that the present results are not due to a startle response [19]–[21], since this effect should have been quite similar in both experiments.

Thus, taken together these findings show first, that hand-centred PPS representation modulates the excitability of the hand corticospinal motor representation, and second, that such modulation acts with a definite time-course. An auditory stimulus presented within the peri-hand space enhances motor system excitability in a very short time window, whereas, in a later time window, a far sound has a greater facilitatory effect than a near sound. These findings are strongly related to each other and can be interpreted in the light of the view that PPS ultimately has a motor function [12], [27].

In monkeys, bimodal neurons representing PPS were first described in the ventral premotor cortex, specifically in area F4 [1], [2], [28], [29], which contains neurons representing specific body parts movements [30]–[32]. Electrical stimulation of such portions of the monkey VPM cortex results in complex motor acts, basically consisting of defensive behaviours [5], [12], [33]. Bimodal neurons are also present in area VIP [3], [9], [34], [35], which is largely interconnected with VPM cortex [36], and electrical stimulation of VIP also results in defensive motor behaviours. Thus, the very same areas integrating multisensory information in a limited space around given body parts also underlie the motor responses of those body parts, meaning that sensory representations of space and motor representations of action overlap in the monkey's bimodal regions. The findings of the present study, which demonstrate that an auditory representation of PPS around the hand results in an immediate modulation of the motor representation of the hand, suggest that a similar overlap between action and spatial processing exists in the human brain as well.

In humans, neural clusters in the ventral premotor cortex and in the inferior parietal sulcus (IPS) have been shown to be more strongly activated when visual or auditory stimuli approach the hand [10] or the face [9]. These areas are likely to underlie PPS representation in humans and may functionally [9] and anatomically [37] correspond to the VPM and VIP areas in the monkey [31]. Moreover, human VPM and IPS are involved in sound localization [38] and motor planning [39]–[40]. Importantly, TMS studies indicate that these areas exert action-related facilitatory influence on corticospinal excitability [40]–[43].

We posit that the fronto-parietal network involved in multisensory integration may be the origin of the modulation of corticospinal excitability found in the present study. The pattern of connectivity of the monkey brain also supports this view. VPM and VIP cortices are strongly interconnected with each other [44] and contain a high number of cells responding to auditory stimuli with early latency of response (10–40 ms) [29], [45]. VPM sends direct connections to the primary motor cortex [46] and also direct connections to the spinal cord [47]. Electrical micro-stimulation of VPM and VIP neurons evokes motor responses with short latency (between 10 and 100 ms) [5], [12], [47]. Therefore, this pattern of fast connectivity would account for the increase of hand motor excitability found in our study 50 ms after the presentation of sounds near the hand. The early facilitation of motor cortex for near, but not far, auditory stimuli may have the function of preparing an immediate motor response for stimuli occurring within the PPS.

Fast sensory-motor transformations should apply to near stimuli potentially requiring an urgent motor reaction, whereas a far stimulus could in principle be processed at later stages and thus may later affect the motor system. We found that the specific MEP enhancement for near sounds disappeared 100 and 200 ms after sound onset, and that at 300 ms the effect fully reversed, so that far auditory stimuli were associated with motor facilitation. At that time delay, auditory stimuli near the hand are likely to be fully processed and evaluated as irrelevant to the body, at least when auditory stimuli carry no consequences, as in our experimental conditions. In contrast, a stimulus in far space is *potentially* relevant for the body at 300 ms, since external objects often move through space. As a consequence, 300 ms after onset, the far stimulus might potentially require a motor response and thus be associated with higher MEPs. The location of an external stimulus in space is not fixed, but varies *in time* as the subject and the external objects move relative to each other. The time-dependent modulation of corticospinal excitability due to near and far stimuli found in the present study captures this relationship between space and time in PPS representation.

We are aware that the effect reported in the present study has been obtained using static sounds, whereas, in everyday life, subjects face with moving stimuli, approaching or receding from the body. Future experiments are needed to explore the relationship between PPS representations and motor responses in more ecological conditions. It should be noted, however, that static stimuli allowed us to describe the time-course of the effect under more controlled experimental conditions. This information is critical to investigate the properties of moving sounds critical for activating PPS representations.

Two more issues need to be discussed before concluding. First, such space and time dependent MEP modulation was present when TMS pulses were delivered at 120% rMT but not at a higher intensity (140% rMT). These results are in keeping with previous findings showing that MEP modulation contingent upon the perception of tactile stimuli is stronger at low than at high TMS intensities [15]. High intensity TMS pulses delivered to the motor cortex hand area are known to recruit less excitable corticospinal neurons within the motor hand area and/or neurons spatially further from the hand area [16], [49]. Our data suggest that these neurons are less affected by the near-far modulation; it is possible that the excitation of such neural populations induced by 140% rMT pulses may have masked the activity of low-threshold motor neurons. Our findings confirm that lower TMS intensities are particularly adept to disclose sensori-motor integrative effects in the human corticospinal system [15].

Finally, near and far auditory stimuli exerted comparable influence on MEPs recorded from the ADM and the FDI muscle, although in our experimental setup the former was closer to the near sound than the latter. The lack of a difference for the effects on these two muscles is not surprising considering that most of bimodal neurons in VPM normally have large RF covering the whole hand [1], [2]. Furthermore, electrical stimulation of VPM bimodal neurons results in complex movements of the hand and the arm, and not in contraction of single muscles.

In conclusion, our findings suggest that in humans, as in monkeys, the representation of the PPS has an immediate effect on the motor system. Processing a stimulus close to the body can result directly in motor preparation. Stimulus distance is defined in a body part-centred reference frame. The effect of PPS representation on the motor system takes into account that spatial relationships between an external stimulus and the subject's body vary in time. These findings support the view that (multi)sensory and motor representations overlap in PPS and suggests that spatial representations are strongly bound up with temporal representations.
